# Primary Renal Synovial Sarcoma Presenting as Haemorrhagic Shock: A Rare Presentation

**DOI:** 10.15586/jkcvhl.v8i3.187

**Published:** 2021-09-03

**Authors:** Ibrahim Alzahrani, Nasser Albqami, Abdullah Alkhayal, Nourah AlOudah, Ali Alyami

**Affiliations:** 1 Urology Department, Security Forces Hospital, Riyadh, Saudi Arabia;; 2 Division of Urology, Department of Surgery, Ministry of the National Guard – Health Affairs, King Abdulaziz Medical City, Riyadh, Saudi Arabia;; 3 Division of Anatomical Pathology, Department of Pathology and Laboratory Medicine, Ministry of the National Guard – Health Affairs, King Abdulaziz Medical City, Riyadh, Saudi Arabia;; 4 King Abdullah International Medical Research Center, Riyadh, Saudi Arabia

**Keywords:** primary renal synovial sarcoma, rare renal tumours, renal tumour histology, soft-tissue sarcomas

## Abstract

Primary synovial sarcoma (PSS) of the kidney is considered the rarest type of all renal sarcomas with specific chromosomal translocation t (X; 18) (p11.2; q11.2). We report the case of a 65-year-old man with no medical conditions who presented to the emergency department with sudden severe right flank pain associated with haemodynamic instability and haemorrhagic shock. Computed tomography (CT) of the abdomen and pelvis revealed a right renal mass. A right open radical nephrectomy was performed. Histopathology revealed a monophasic synovial sarcoma. The patient received six cycles of docetaxel and gemcitabine as adjuvant chemotherapy. No sign of recurrence was seen on a follow-up CT urogram. This rare tumour often presents atypically, and clear guidelines regarding appropriate treatment are lacking. Our case showed that treatment with docetaxel/gemcitabine after an open radical nephrectomy is promising.

## Introduction

Primary synovial sarcoma (PSS) of the kidney is a type of renal sarcoma and is considered the rarest of all renal sarcomas (1). Leiomyosarcoma accounts for the majority of renal sarcomas (50%–60%), followed by liposarcoma (10%–15%). Additional histological subtypes include osteogenic sarcoma, chondrosarcoma, rhabdomyosarcoma, fibrosarcoma, malignant fibrous histiocytoma, angiosarcoma, anaplastic sarcoma and Ewing’s sarcoma (2). PSS of the kidney was first described in 1999 by Faria (3). In general, it accounts for less than 1% of all renal malignancies. PSS usually affects younger individuals of both sexes, aged between 20 and 50 years (1). Diagnosis is often difficult because there are no specific clinical or imaging characteristics associated with PSS (1). PSS of the kidney presents similarly to other renal tumours without specific clinical signs. Moreover, there are no imaging characteristics that confirm the diagnosis of synovial sarcoma (1, 4, 5). Histologically, renal synovial sarcoma can be classified as a biphasic synovial sarcoma, monophasic spindle synovial sarcoma and monophasic epithelial synovial sarcoma (6, 7). These tumours have a specific chromosomal translocation t (X; 18) (p11.2; q11.2), resulting in the fusion of the synovial sarcoma genes *SYT-SSX1* and *SYT-SSX2*, and rarely, *SYT-SSX4* (6, 7). To date, there is no standard treatment protocol for synovial sarcomas (8). Primary surgical treatment is currently the treatment of choice, although surgery alone has a poor prognosis. The use of adjuvant chemotherapy regimens involving ifosfamide and adriamycin has also been reported; however, no clear guidelines outlining the appropriate treatment of this rare tumour (8) are available. The most recent systemic review included 96 studies with 185 cases of PSS of the kidney and showed that surgery, with adjuvant chemotherapy, was the most common treatment. The ifosfamide-based treatment was most frequently used, combined with doxorubicin or epirubicin (9). In the present case, a man who presented with bleeding PSS of the kidney was successfully treated by open radical nephrectomy followed by a docetaxel/gemcitabine adjuvant chemotherapy.

## Case Report

**Figure 1:**
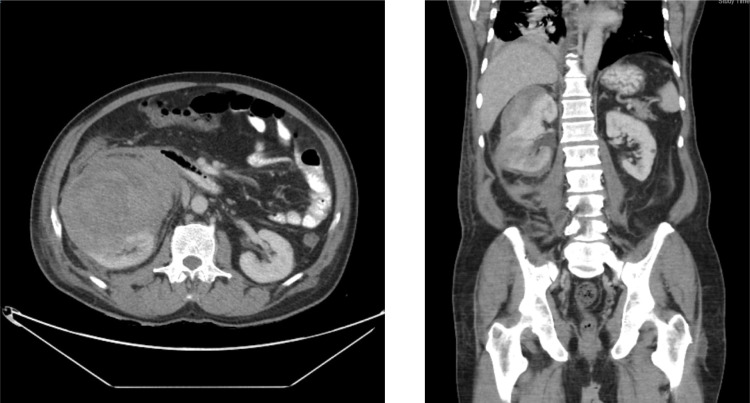
A CT scan of the abdomen and pelvis shows 10 × 11.2 × 18.7 cm right large subcapsular, perirenal and retroperitoneal haematoma compressing and exerting mass effect on the kidney with a cortical defect at the lower pole with adjacent 2 cm dense foci.

**Figure 2:**
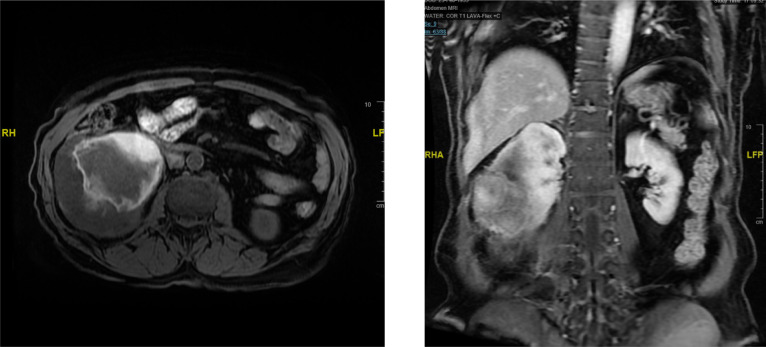
MRI of the abdomen shows there is a completely exophytic 3.8 × 8.5 cm soft tissue mass arising from the right inferolateral renal cortex and confined to the perinephric space with new angiogenesis without sinus fat or collecting system invasion, and no regional lymph nodes or renal vein invasion. No intra-abdominal metastasis.

**Figure 3:**
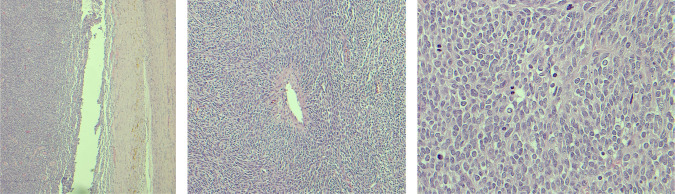
The histopathology sections show an infiltrative spindle cell tumour with haemangiopericytoma-like blood vessels with little intervening stroma arranged in fascicular and storiform patterns. The cells are monotonous with scant amphophilic cytoplasm, ovoid to spindled vesicular nuclei with evenly dispersed chromatin and inconspicuous nuclei with mitotic rate of 3/HPF.

**Figure 4:**
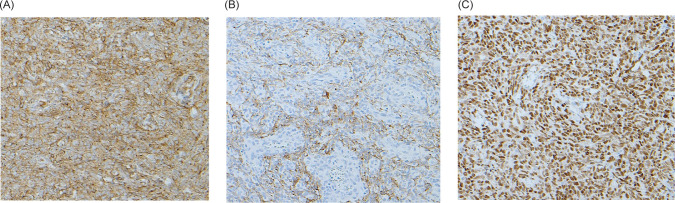
The immunohistochemistry of the tumour tissue. (A) The positive reaction to CD99. (B) The positive reaction to pan-CK. (CD) The positive reaction to TLE-1.

A 65-year-old man with no medical conditions presented to the emergency department with sudden severe right flank pain associated with haemodynamic instability. A computed tomography (CT) scan of the abdomen and pelvis showed a 10 × 11.2 × 18.7 cm right large subcapsular, perirenal, and retroperitoneal haematoma compressing and exerting a mass effect on the kidney with a cortical defect at the lower pole with adjacent 2 cm dense foci (Figure 1). Resuscitation was performed, and bleeding was controlled by embolisation. Magnetic resonance imaging (MRI) of the abdomen showed a 3.8 × 8.5 cm soft tissue mass arising from the right inferiolateral renal cortex and confined to the perinephric space with new angiogenesis without sinus fat or collecting system invasion, and no regional lymph node or renal vein invasion. No intra-abdominal metastasis (T3-N0-M0) was observed (Figure 2). The patient underwent a right open radical nephrectomy. The nephrectomy specimen weighed 1106 g and measured 18.0 × 11.5 × 9.0 cm. Histopathological examination revealed an infiltrative spindle cell tumour with haemangiopericytoma-like blood vessels with little intervening stroma arranged in fascicular and storiform patterns (Figure 3). On immunohistochemistry analysis, the cells were positive for vimentin, CD99, TLE-1 and BCL2, with scattered positivity for pan-CK, and negative for CD34, CD31, RCC, CD10, S100, EMA and SMA. This is consistent with the diagnosis of synovial sarcoma, monophasic type (Figure 4). An oncology evaluation was performed, and the patient was started on chemotherapy with six cycles of a docetaxel and gemcitabine combination. The plan was to follow up the patient for one year with a CT scan of the abdomen and pelvis. After nine months from the surgery, a CT urogram showed no signs of recurrence.

## Discussion

Soft-tissue sarcomas are considered a rare type of cancer that accounts for only about 1% of all cancers. Approximately, 8700 new cases are diagnosed each year in the United States (6). The World Health Organization has defined approximately 50 tumour subtypes relevant to soft-tissue sarcomas, which are generally named according to the tissue they most closely resemble (6). Synovial sarcoma accounts for 5%–10% of soft-tissue sarcomas (4). This tumour can arise anywhere in the body, and the most common locations are in the limb or limb-girdle or within the abdomen (retroperitoneal or visceral and intraperitoneal) (6, 10). Generally, it affects younger individuals of both sexes, aged between 20 and 50 years, with a median age of 35 years (1, 4, 5). Blas and Roberti (2021) reviewed 96 studies, including 185 clinical cases of PSS of the kidney, and found that the predominant location of the tumour was the right kidney. Surgery was the treatment of choice, with adjuvant chemotherapy; most frequently ifosfamide-based, combined with doxorubicin or epirubicin (9). The median survival time was 34 months, with a mortality rate of 29% and a recurrence rate of 39.8% (9). In our report, the patient underwent a right open radical nephrectomy with six cycles of docetaxel and gemcitabine as adjuvant chemotherapy. Regular follow-up with a CT urogram showed no signs of recurrence.

## Conclusions

Primary synovial sarcoma of the kidney is rare and accounts for less than 1% of all renal tumours. This rare tumour can present as a ruptured mass with haemorrhagic shock. Although no clear treatment guidelines are available for this tumour. This case study reported promising results using a docetaxel/gemcitabine combination treatment strategy.
